# Simulating Precision Feeding of High-Concentrate Diets with High-Fat Inclusion and Different Plant-Based Saturated, Unsaturated, and Animal Fat Sources in Continuous Culture Fermenters

**DOI:** 10.3390/ani15162406

**Published:** 2025-08-16

**Authors:** Saad M. Hussein, Thomas C. Jenkins, Matias J. Aguerre, William C. Bridges, Gustavo J. Lascano

**Affiliations:** 1 Department of Animal and Veterinary Sciences, Clemson University, Clemson, SC 29634, USA; tjnkns@clemson.edu (T.C.J.); maguerr@clemson.edu (M.J.A.); 2 Department of Animal Production, College of Agriculture, University of Kirkuk, Kirkuk 36001, Iraq; saad.hussein@uokirkuk.edu.iq; 3 School of Mathematics and Statistical Sciences, Clemson University, Clemson, SC 29634, USA; wbrdgs@clemson.edu

**Keywords:** continuous culture, precision feeding, high fat, high concentrate, animal fat

## Abstract

Controlling dry matter intake (DMI) is one strategy to reduce feed costs and increase efficiency. Including fat at a high concentrate level can increase the energy density of diets fed to ruminants, thus reducing DMI further. Therefore, the objective of this study was to evaluate the effects on fermentation and nutrient digestion of including different fat sources when high-concentrate diets with high-fat inclusion are used under simulating precision feeding in continuous culture. We hypothesized that incorporating different fat sources into the aforementioned program can improve nutrient utilization without affecting rumen fermentation. Four treatments were randomly assigned to eight continuous cultures in a randomized complete block design and ran for two periods of 10 d. Diets included a high concentrate level (HC; 65% DM) with high-fat inclusion starting with a 3% basal level of fat in the diet as the control (0% added fat; CON) and 9% fat in the diet (6% added poultry fat, PF; 6% added coconut oil, CO; and (6% added soybean oil, SO). Data were analyzed using the MIXED procedure of SAS with repeated measures. The DM, OM, NDF, and ADF digestibility coefficients (dCs) were higher for PF and CO, followed by SO and then CON. Starch and FA dCs were higher for different fat sources than for the CON. The total VFA concentration was higher for CON. There was a reduction in acetate and propionate with different fat sources. The mean culture pH and NH_3_N were the highest for CO, followed by PF, then SO, and CON. The protozoa population was higher for CON than for the other fat treatments, followed by CO, PF, and SO. These results suggest that simulated precision feeding using continuous culture fermenters with high-concentrate diets up to 65% and high fat up to 6% can improve nutrient digestion approximately to 15% with changes in fermentation rate and profiles.

## Simple Summary

Including saturated fatty acids in high-concentrate diets can further decrease dry matter inputs without negatively affecting the digestibility and nutrient utilization in simulating precision feeding programs using continuous culture fermenters. This experiment reports that poultry fat and coconut oil can be included in the rations up to 6% under continuous culture conditions in order to reduce the amount of intake and reduce nutrient loss.

## 1. Introduction

High-fiber-based diets are inefficient in terms of energy and protein utilization and lower digestibility compared to concentrates [1,2]. However, that can be potentially enhanced by incorporating concentrate, fat, or both to make the diets more energy dense [3,4]. Diets used for precision feeding are more nutrient dense, allowing for an increase in energy and nutrient utilization efficiency while decreasing nutrient loss. Manipulating nutrient fractions allows precision feeding to achieve adequate nourishment [5]. Including dietary fat can increase the energy density of diets, reducing intake further. The use of high-concentrate precision-fed diets showed improvement in OM digestibility [5] and resulted in similar effects on rumen fermentation [6,7]. However, in addition to the increase in grain costs, negative effects of high-level feeding of concentrates on dairy cattle’s fiber intake and acidosis incidence can occur [8,9]. Feeding fat has gained interest in the last few decades. Adding fat to dairy diets became common practice for its potential to increase the energy density in diets, improve palatability, and reduce feed dustiness [10]. Unsaturated fatty acids, as in soybean oil (SO), can decrease fiber digestion [11,12], whereas saturated medium-chain fatty acids, as in coconut oil (CO), may improve rumen fermentation [4,13,14]. Additionally, cost-effective by-products from numerous industries, such as the poultry industry, can be utilized by ruminants. Poultry fat (PF) is a by-product of chicken processing and is extensively produced worldwide as a potential source of energy [15,16,17].

On the other hand, the total dietary fat should not exceed 6–7% of the dry matter intake (DMI), and traditional dairy heifer diets typically contain between 2 to 3% fat [18]. In a study conducted by [19], the dietary fat reached up to 7% when fed high fat from non-defatted dried distillers’ grains (DDGS) to dairy heifers. Also, in an earlier study by [20], the diet’s fat was close to 5% when a large portion of the heifer diet was supplied by wet distillers’ grains and soybean hulls.

The level of fat saturation showed an effect on digestibility and fermentation. In a study conducted by [21] on the effects of saturation of fat sources in steers, they reported that increasing fat sources’ saturation tended to increase the NDF and ADF digestibility in the rumen. Other studies have reported no differences in ruminal or total tract digestibility of OM or fiber in lactating cows fed diets with increasing amounts of dietary fat or different sources of fat [22,23,24,25]. A study by [26] reported that acetate responded quadratically as the fat sources’ unsaturation degree increased. In addition, ref. [21] reported a decrease in acetate’s molar proportion when different saturation fat was fed and increased linearly as saturation increased. Furthermore, in a recent study by [4], they reported higher nutrient digestion and fermentation profile when using CO and PF sources added up to 6% supplementary fat to low-forage diets using an in vitro gas production model. The objective of this study was to evaluate the effects on fermentation and nutrient digestion of including different unsaturated fat sources when high-concentrate diets with high-fat inclusion are used when simulating precision feeding in continuous culture. We hypothesized that incorporating different fat sources in a high-concentrate diet into the aforementioned program can improve nutrient utilization without compromising fermentation and fermenters’ digestibility.

## 2. Materials and Methods

### 2.1. Treatments and Experimental Design

Diets included a high concentrate level (HC; 65% DM) with high-fat inclusion starting with a 3% basal level of fat in the diet as the control (0% added fat; CON) and 9% fat in the diet: (6% added poultry fat, PF; stabilized poultry fat; Valley proteins, Inc., Ward, SC, USA), (6% added soybean oil, SO; pure soybean oil; Nature’s oil, Streetsboro, OH, USA), and (6% added coconut oil, CO; fractionated coconut oil; Nature’s oil, Streetsboro, OH, USA). The experiment was designed as a randomized complete block design consisting of 4 experimental diets replicated within 2 blocks of 4 dual-flow continuous culture fermenters during 2 periods of 10 d with 4 replicates per treatment. Each period started with a clean fermenter and was inoculated with ruminal contents collected from 2 cannulated Holstein cows fed a 50% forage/50% concentrate diet. Adaptation to treatment rations was made over the first 7 d of each period and 3 d for sampling collection. Treatments were randomly assigned to one of 4 continuous culture fermenters in each block. All diets were fed to the fermenters as total mixed rations (TMRs) and predicted nutrient composition was determined using [18]. Diets were formulated to simulate a precision feeding program in continuous culture fermenters to restrict intake and also to provide equal amounts of ME and N to supply 1.70 g N/kg BW^0.75^ in Holstein heifers, which has been observed to maximize N utilization and allow for 800 g/d of ADG [5,27]. The dietary ingredients, chemical composition, and fatty acid profile are presented in Table 1. Fermenters receiving the CON treatment were fed a greater amount of TMR [(CON; 3% fat; 53.4 g/d as fed)] than the other treatments [(PF; 9% fat; 47.7 g/d); (SO; 9% fat; 47.7 g/d); (CO; 9% fat; 47.7 g/d as fed)]. That was because of the different energy concentrations of the diets and different levels of fat inclusion between the control and the other treatments required to maintain isocaloric intake. Rations were prepared, mixed in advance, split into 2 equal amounts, and fed to the continuous culture fermenters daily at 0900 and 2100 h.

### 2.2. Continuous Culture Conditions

All procedures involving the surgical and animal care protocols were approved by the Clemson University Institutional Animal Care and Use Committee (AUP2019-074). Around 1800 h, rumen contents were collected from two rumen-cannulated Holstein cows fed a 50% forage/50% concentrate diet and strained through two layers of cheesecloth into a prewarmed sealed container. The filtered rumen fluid was combined from both cows, mixed with a buffer at a 1:1 ratio according to the methods of [28], and purged with CO_2_ until inoculation into the continuous culture fermenters. Moreover, the time from inoculum collection to fermenter inoculation did not exceed 60 min. Approximately 750 mL of diluted inoculum was added to each dual-flow fermenter. The fermenters’ design and operation were based on a previous design outlined by [29], with some modifications including the use of an overflow sidearm that angled downward at approximately 45° to facilitate emptying. In addition, a faster stirring rate (45 rpm) still allowed for the stratification of particles into three layers: an upper mat layer, a middle liquid layer of small feed particles, and a lower layer of dense particles [30]. A higher feeding rate for the control treatment (53.4 g/d as fed; 26.7 g/feeding) and a lower feeding rate in the other treatments to simulate restricted intake were utilized. The buffer solution was also delivered continuously to the cultures using a peristaltic pump. Also, it was manipulated to achieve different liquid and solid passage rates [kpl; 8.6%/h for CON and 7.7%/h for other treatments); (kps; 3.8%/h for CON and 3.2%/h for other treatments; Appendix A)] to simulate a precision feeding program in dairy heifers based on an in vivo study [31]. The buffer solution was used to dilute the inoculum [28] at a 1:1 ratio and was selected based on previous works in our lab and included a greater level of NaHCO_3_ to maintain culture pH. The cultures were maintained for 10 d: 7 d for adaptation duration to obtain a steady-state fermentation in the cultures and 3 d for culture sampling [30]. A study by [32] reported that the cultures’ microbial population requires a 5 d adaptation period. These durations are commonly used in continuous culture experiments [33,34,35]. The fermenters’ temperature was maintained at 39 °C using a recirculating water bath. Each fermenter was continuously purged with CO_2_ at a rate of 20 mL/min to maintain anaerobic conditions, and gas flow rates were checked before the morning and evening feedings to ensure consistency. The culture’s pH was monitored using handheld pH probes and calibrated at the start of each period. Oxidation–reduction potential (E*h*) was measured using the redox probe (Traceable 4277 pH/ORP Meter, Control Company, Webster, TX, USA) during the sampling day at the same time points of pH measuring. The relative hydrogen score (rH) was calculated using the Clark equation for deriving the rH from the pH and E*h*.

### 2.3. Sample Collection and Analysis

On d 8, 9, and 10 of each period, liquid and solid digesta overflows from each fermenter were collected in a 2 L Erlenmeyer flask immersed and covered in an ice bath to stop the microbial activity. The overflow flasks were weighed, and the total volume was recorded once daily at 2030 h. A 20% aliquot of the overflow was collected in a pre-labeled container and immediately frozen at −20 °C. The 3 d composited overflow samples were later thawed, homogenized, and subsampled for later analysis of DM, OM, NDF, ADF, and LCFA. On the last day (d 10) of each period, cultural contents were mixed thoroughly (120 rpm) during sampling to ensure an adequate sample from the cultures. The culture pH and E*h* were measured and recorded at 0 (before feeding), 2, 4, 6, 8, 10, and 12 h, and a 5 mL sample of culture contents was taken at the same time points for protozoa (kept in the fridge at 4 °C), VFA, and ammonia analysis (frozen at −20 °C).

Feed and dried overflow samples were ground using a Wiley Mill (Arthur H. Thomas Co., Philadelphia, PA, USA) through a 2 mm sieve and analyzed for DM, OM, ash, and EE [36], as well as through a 1 mm sieve for NDF and ADF [37] using an ANKOM200 Fiber Analyzer (ANKOM Technology Corporation, Fairport, NY, USA) with heat resistant α-amylase and sodium sulfite utilized in the NDF procedure. Starch was analyzed on reground samples (<0.5-mm screen) using an enzymatic procedure [38]. Culture samples (5 mL) were pipetted to 15 mL centrifuge tubes containing 1 mL of metaphosphoric acid (25%; *w*/*v*), and then these tubes were stored at −20 °C until VFA and ammonia analysis [39]. Samples were later thawed and centrifuged at 40,000× *g* for 30 min at 4 °C. After centrifugation, 1 mL of the supernatant was placed in a 2 mL Eppendorf microcentrifuge tube and used for the analysis of NH_3_N according to the methods of [40], with modifications, including reduced sample and reagent volumes to accommodate the use of a 96-well plate reader. Another 0.5 mL of the supernatant was combined with 0.5 mL of distilled water and 100 μL of internal standard (86 μmol of 2-ethylbutyric acid/mL) in a GC vial.

Samples for VFA were then analyzed by GC–flame ionization detection according to the methods of [41] and injected into a Hewlett-Packard 6890 gas chromatograph (San Jose, CA, USA) equipped with a custom packed column (2 m × 0.32 cm × 2.1 mm ss; 10% SP-1200/1% H3PO4 on 80/100 Chromosorb WAW). Additionally, a 4 mL culture sample was pipetted and preserved in 4 mL of methyl green formalin–saline solution (1:2 dilution) and stored in darkness at 4 °C for protozoa counting [42]. Dried ground feed and overflow samples were sent to the Multi-User Analytical Laboratory and Metabolomics Core, Clemson University, SC, for the LCFA analysis. Quantities of individual fatty acids present in the cultures were determined on a Shimadzu GC-2010 gas chromatograph with a flame ionization detector (Shimadzu Corporation, Kyoto, Japan). It was equipped with an SLB-IL111 (Sigma, St. Louis, MO, USA) fused silica capillary column (L × I. D. 100 m × 0.25 mm) with 0.2 um film thickness. The initial temperature was held at 140 °C for 3 min then increased by 3.7 °C per min up to 220 °C for 60 min. The carrier gas was helium purged at 20 cm/s. Fatty acid peaks were identified and separated by comparison of the retention times to known standards.

### 2.4. Calculations and Statistical Analysis

Fractional passage rates were calculated according to [31] as follows:

The liquid passage rate in the in vivo study [31] was 8.93%/h for LF (45% forage). Therefore, we assumed 8.60%/h would be the control diet’s liquid passage rate (35% forage) in our study.

The liquid passage rate was decreased based on the decreased dry matter intake as we increased the fat inclusion in the diets.

Liquid passage rate (%/h) = dry matter intake (g/d) × liquid passage rate for the control (mL/h) × dry matter intake for the control (g/d),

Buffer input (mL/h) was calculated as follows:

Buffer input (mL/h) = liquid passage rate (%/h) × fermenter volume (mL),

In the same way, the solid passage rate was calculated and based on the results of our study.

Metabolizable energy intake (Mcal/d) was calculated as follows:

ME (Mcal/d) = (digested OM intake × 4.409 (Mcal/Kg) × 1.01 − 0.45) × 0.82,

assuming that digestible OM intake and total digestible nutrient intake were equal.

That equation was used for the control diet, which was modified from [18]. To better represent the increase in energy as fat increased in the diets, another modified equation from [18] was used as follows:

ME (Mcal/d) = (digested OM intake × 4.409 (Mcal/Kg) × 1.01 − 0.45) + (0.0046 × (EE − 3) × 0.82).

All statistical analyses were conducted in SAS version 9.4 for Windows (SAS Institute Inc., Cary, NC, USA) using the MIXED procedure. Data were analyzed as a randomized complete block design with period and fats as a fixed effect, fermenters as a random effect, and repeated measures as needed (Ammonia, VFA, protozoa, E*h*, and pH) for the following model:

Yijk = μ + Fi + Pj + Ck + eijk,

where Yijk = the dependent variable, μ = the overall mean, Fi = the fixed effect of fat, Pj = the fixed effect of the period, Ck = the random effect of the fermenters within blocks (k = 1 to 8), and eijk = the residual error. The PDIFF option adjusted by the Tukey method was included in the LSMEANS statement to account for multiple comparisons. For observations where multiple repeated measures occurred in a period, the fixed effects of time and its interaction with other fixed effects were included in the model based on a repeated measures analysis [43]. Covariance structures of simple, autoregressive, or compound symmetry were chosen for use in the repeated measures analysis based on the lowest values of Akaike’s Information Criterion and Schwartz’s Bayesian Criterion. Residuals for all models were found to be normally distributed (Shapiro–Wilk test for normality). Least square means are presented in tables, and evidence for statistical significance was declared at *p* ≤ 0.05, while trends for main effects and interactions are discussed at 0.05 < *p* ≤ 0.10.

## 3. Results and Discussion

### 3.1. Diet Composition and Nutrient Inputs

The diet ingredients, chemical composition, and fatty acid profile are presented in Table 1. The dietary EE concentrations increased in the diets up to 9% with the inclusion of different lipid sources and, consequently, the ME concentration; therefore, the daily feeding amount decreased as different lipid sources increased. The addition of different lipid sources to the diets resulted in two different proportions of FA concentrations in the diets, and its input increased as well. As planned, the fat inclusion replaced the ground corn in the control diet, resulting in a decrease in starch and NFC in the other three different fat treatments. All other components of the rations were formulated to be similar between treatments.

Daily starch and NFC inputs were decreased as fat was included in the diets and were the opposite with EE input as it increased to achieve the planned diets. Consequently, there was an input fat effect on OM, NDF, ADF, starch, and NFC to maintain the isoenergetic and isonitrogenous treatment design. The liquid and solid passage rates were lower for different fat-fed fermenters compared to the control-fed fermenter (Appendix A). Passage rates of diets can be slower when intake is limited [44,45], and we expected to be even slower when fat is added to the diets.

### 3.2. Digestibility of Nutrients

**Figure 1:**
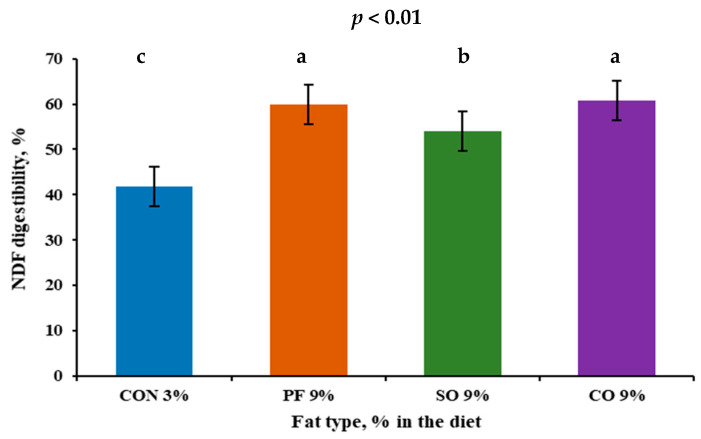
Neutral detergent fiber apparent digestibility of continuous culture fermenters fed high-concentrate diets (HC 65%) with high-fat inclusion and different lipid sources (CON 3%, PF 9%, SO 9%, and CO 9% DM). Means in the same row, followed by different superscripts (^a^, ^b^, and ^c^), are significantly different (*p* < 0.05).

Apparent digestibility coefficients (dCs) are outlined in Table 2. The dCs of DM, OM, NDF (Figure 1), and ADF were greater for CO-fed fermenter and PF-fed, fermenter followed by SO-fed fermenter and then CON-fed fermenter. These observations are consistent with results on the effects of saturation of fat sources in steers [21]; it has been reported that increasing the saturation of fat sources tended to increase the NDF and ADF digestibility in the rumen. Several other studies have reported no differences in ruminal or total tract digestibility of OM or fiber in lactating cows fed diets with increasing amounts of dietary fat (up to 5.7% total FA ~7% EE) or different sources of fat [22,23,24,25]. In contrast, it has been reported in a meta-analysis [46] that adding 3% of saturated fats or calcium salts to the diets increased total-tract NDF digestibility. However, medium-chain fats and unsaturated vegetable oil decreased total-tract NDF digestibility of lactating dairy cows. It has been reported, using in vitro models, that CO up to 6%, which contains a high saturated medium-chain fatty acid, had no adverse effects on DM digestibility in the in vitro gas fermentation production technique [4,47]. In a study conducted by [19], the authors reported a higher dC for NDF and ADF when heifers were limit fed high-fat DDGS (7.00% EE) compared to low-fat DDGS (3.08% EE), whereas the DM and OM did not differ between the treatments. It was suggested that the high-fat DDGS diet contains a lower starch content compared to the low-fat DDGS, which is the case in our study (Table 1), which resulted in a higher pH and efficiency of utilization of fiber and improved the total-tract digestion. Also, ref. [48] observed a quadratic DM, OM, NDF, and ADF dC response to increasing levels of DDGS up to 14% inclusion in the diets (4.99% total FA ~6% EE). These results did not agree with a study conducted using two levels of fat with no added fat [30] or 3.3% added SO in continuous culture fermenter where they did not observe any effects on DM and ADF dCs between the two levels of fat in the diets. In the present experiment, fat represented 3% and 9% of the diets, respectively, which is larger than what has been reported in the above study. Depression in DM, OM, NDF, and ADF dCs when continuous culture fermenters were fed high SO compared to low SO were observed [30]. Also, these authors stated that the dietary polyunsaturated fatty acids had been shown to depress the fiber dC by limiting the growth of fiber digestion bacteria [49], and this finding is common in the literature [50]. However, no effects on the dC when heifers were limit fed DDGS with ad libitum grass hay were reported [51]. They related that to feeding grass hay ad libitum, which resulted in a slightly different limit feed program than the typical one. Also, the passage rates of diets can be slower when intake is limited [44,45], and we expected them to be even slower as fat was added to the diets, as we have it in the current study (Appendix A).

**Figure 2:**
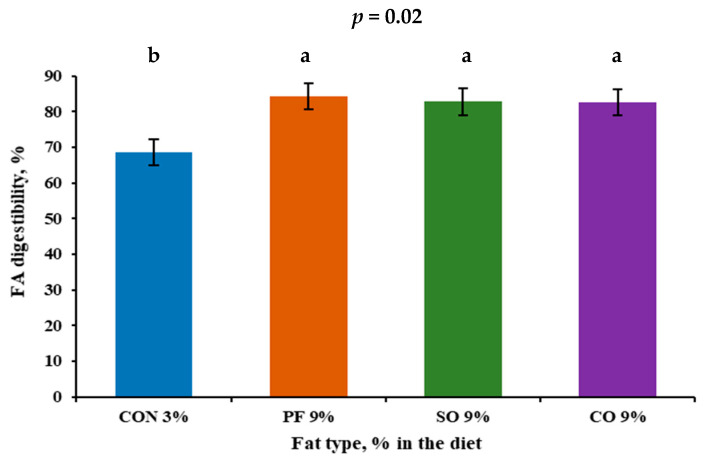
Fatty acids apparent digestibility of continuous culture fermenters fed high-concentrate diets (HC 65%) with high-fat inclusion and different lipid sources (CON 3%, PF 9%, SO 9%, and CO 9% DM). Means in the same row, followed by different superscripts (^a^, and ^b^), are significantly different (*p* < 0.05).

The greater digestibility of fat-fed fermenter can be attributed to the greater digestibility of the ingredients in these diets and also to a higher retention time of these diets in the culture fermenter [45,52] as we decreased the passage rate with lower intake, as we planned in our study. On the other hand, the lower NDF and ADF dCs in the CON-fed fermenter could be related to the availability of rapidly fermented ingredients such as starch and NFC (Table 1). That could also be related to more numerous amylolytic bacteria populations associated with CON diets [53]. Furthermore, this could be attributed to the lower pH level for the CON-fed fermenter because cellulolytic bacteria are very sensitive to pH, and their activity and growth start to decline under pH 6.0 [54]. The FA (Figure 2) and starch dCs were higher in the fat inclusion-fed fermenters than the CON-fed fermenter. That is mainly because of the lower starch and NFC contents (Table 1) and their inputs (Appendix A) as corn was replaced with fat in the diets. Also, the lower passage rate and higher retention time resulted in more efficient fat and starch utilization in the continuous culture fermenter system.

### 3.3. Fatty Acid Flows and Biohydrogenation

The overflows of major fatty acids are detailed in Table 3. The inclusion of CO showed an increase in the overflow of individual saturated FAs C12 and C14. That is mainly because the CO is relatively high in saturated medium-chain fatty acids such as C12 and C14, as we can see from Table 1. That agrees with a study conducted by [55] using continuous culture fermenters and different fat supplements. They observed that the C14 flow was the highest when fish oil was fed, which is relatively higher in C14 compared to animal fat (RumoFat) and SO, whereas the overflow of saturated FAs C16, C18, and C22 was the highest with PF inclusion. Similarly, this can be attributed to PF high saturated long-chain fatty acids such as C16 and C18, as shown in Table 1 and their inputs in Appendix A. These observations also agree with an in vivo study where animal fat (RumoFat) showed the highest C18 flow [55]. A study conducted by [30] observed a reduction in saturated FAs C12, C14, C20, C22, and C24 when fermenters were fed, increasing starch degradability. Also, ref. [30] reported increased daily outflows of individual saturated and total fatty acids when fermenters fed high-fat diets compared to low-fat diets. Similarly, in a study conducted on feeding two levels of fat (no added fat or 3.64% of DM) to continuous culture fermenters, the high-fat-fed fermenters showed a higher outflow of C:16, C20, C22, and C24 compared to low-fat-fed fermenters [33].

The SO-fed fermenter showed the highest flow of individual unsaturated FAs C18:1, C18:2, and C18:3. That can be attributed to the fact that the SO is relatively high in unsaturated long-chain fatty acids (Table 1). In an in vitro study, the SO inclusion resulted in the highest flow of C18:1, and they attributed that to the highest proportion of the C18 unsaturated FA, among other treatment diets [55]. Other studies also reported similar increases in C18:1 flows in the rumen [56,57] and duodenum [58] with SO inclusion in the ruminant animal’s diet. The decrease in C18:1 and C18:2 overflows in CO-fed fermenter and PF-fed fermenter is partially related to replacing ground corn with fat and the high biohydrogenation efficiency of high-fat diets [59]. That is in agreement with a study on dry dairy cows fed two levels of crude fat (2.9% and 7.6%) that showed a decrease in the C18:2 FA [60].

Additionally, the CON-fed fermenter showed a lower total fatty acid flow compared to the other treatments. That is due to the higher content of starch and NFC, as well as the lower fat content. An increase in the outflows of C18:2 and C18:3 from the fermenters fed high starch resulted in a lower extent of biohydrogenation [30]. Cultures under low-pH conditions (5.65) showed less disappearance of the C18 unsaturated FA [61]. Most rumen microbial growth and enzyme activities could be impacted under low-rumen-pH conditions [62,63]. In the current study, the lower pH in CON-fed fermenters (Table 4) may have affected culture bacteria and reduced the biohydrogenation rates.

Part of the differences in the unsaturated fatty acids flow is related to the differences in the dietary contribution of C18:1, C18:2, and C18:3, while the other part is related to the rate of biohydrogenation. The biohydrogenation rate of C18:2 was decreased with a CON-fed fermenter. That aligns with our observations with a lower amount of C18:0 flows for the CON-fed fermenter and indicates a reduction in the biohydrogenation pathway to completion at C18:0. Based on PF’s effect on unsaturated FA C18, the PF-fed fermenter showed the highest percentage in the biohydrogenation of C18:2 and C18:3, followed by both of the SO-fed fermenter and CO-fed fermenter and then CON-fed fermenter. These results agree with several in vivo and in vitro studies, as they observed that the biohydrogenation rates of unsaturated fatty acids increased as the inclusion of fat increased in the diets [30,33,60].

### 3.4. Characteristics of Fermentation

The culture VFA profile, NH_3_N, pH, reduction potential (E*h*), relative hydrogen score (rH), and total protozoa counts are shown in Table 4. The inclusion of different lipid sources in the diets decreased the total VFA concentrations with the lowest CO-fed fermenter compared to the CON diet. The total VFA concentration decreased when different fat sources were fed compared to the control diet [21]. This was attributed to the lower fermentable carbohydrate content in fat-fed diets as corn was replaced with fat, as in the current study, to maintain the isocaloric intake. A study conducted by [47] reported a lower total VFA concentration in the in vitro gas production technique and after 48 h of incubation with 5% CO in the diet. They attributed that to the negative effect of medium-chain fatty acids on the fermentation. Medium-chain fatty acids in CO are small enough to penetrate and disrupt the cell membranes by readily dissolving in the lipid phase [64]. Also, it inhibits the enzymes involved in energy production and nutrient transfer, leading to reversible and irreversible changes that could lead to the microbial cell’s death. Also, the higher total VFA concentration for CON-fed fermenters could be related to the pH [65]. In the current study, the pH was the lowest with the CON-fed fermenter than for other treatments. In addition, as DM inputs decrease with fat inclusion, the passage rate decreases and the retention time increases in the continuous culture fermenter as planned in the current study, and that could be the reason behind the lower total VFA as fat increases in the diets (Appendix A). Furthermore, this reduction could be mainly because of the reduction in acetate concentrations as fat included in the diets, specifically with the SO-fed fermenter and CO-fed fermenter. Even though the dCs of NDF and ADF were the highest by CO inclusion, the reduction in fiber intake and starch intake is the reason behind the reduction in acetate concentration [51]. Acetate production within the rumen results from the fermentation of structural carbohydrates by cellulolytic bacteria [66]. It has been reported that the acetate responded quadratically as the fat sources’ unsaturation degree increased [26]. In addition, a decrease in acetate’s molar proportion was observed when a different saturated fat source was fed and increased linearly as saturation increased [21]. Furthermore, acetate results in structural carbohydrate fermentation by cellulolytic bacteria, and these bacteria can be inhibited by lower NDF inputs as in the present study, which may explain the lower acetate concentration for fat-fed fermenter [62]. Rumen fermentation is not affected when fat levels are low in the diets because rumen microbes are able to saturate FAs, but this capacity can be exceeded at higher levels, and FAs can accumulate in the rumen and interfere with rumen fermentation [18].

However, the propionate concentrations were not affected by fat inclusion except in the CO-fed fermenter, which was lower than the CON-fed fermenter. The propionate observation agrees with a study conducted by [47], as they reported a lower propionate concentration in the CO diet after 48 h incubation. Also, this agrees with [26] when Holstein heifers are fed different degree of fat saturation (tallow, partially hydrogenated tallow, and animal–vegetable fat). A linear decrease in propionate as saturation increased was observed, even though the acetate/propionate ratio was not affected and was similar between the treatments [21]. Some studies have reported that feeding fat can decrease the acetate/propionate ration [21,26] or it be unchanged [67]. Butyrate, valerate, and isobutyrate concentrations were lower in the CON-fed fermenter than the CO-fed fermenter but were not different from the PF- and SO-fed fermenters. The reduction in the valerate concentration in the CON-fed fermenter could be related to the higher liquid fraction kp accompanied with a lower retention time for the CON-fed fermenter [32,68]. In the present study, the CON-fed fermenter showed a lower NH_3_N concentration. If energy is available, the AAs can be incorporated into bacteria without deamination [69], which would explain the lower isobutyrate concentration with lower deamination in the CON-fed fermenter compared to the CO-fed fermenter. These results are comparable to those reported by several studies conducted on dairy heifers limit fed DDGS [48,51,70]. In addition, these reports could be due to the decline in the culture bacteria population with fat inclusion [48], and this is supported by the decline in total protozoa counts as fat is included in the diets in the current study. Differences in starch contents and intake can be the reason behind the shift in VFA concentrations and the decrease in acetate and increase in propionate concentrations [51]. Also, the authors suggested that the higher propionate concentration is related to more energy-efficient rumen fermentation in heifers fed DDGS diets [70] because there is less methane and carbon dioxide production in propionate as compared with acetate [71].

The ammonia concentration increased as the different unsaturated fats were included in the diets compared to the CON-fed fermenter, with the highest concentration in the CO-fed fermenter, followed by PF- and then SO-fed fermenters. The lower NH_3_N in the CON-fed fermenter than in fat-fed fermenters could be due to the use of ammonia for the de novo synthesis of AAs. Studies conducted by [19,48,70] observed similar results, and they attributed that to the high-fat DDGS diet containing a lower starch content compared to the low-fat DDGS, which is the case in our study (Table 1); therefore, the microbial capacity to assimilate amino acids and ammonia was negatively affected, and NH_3_ accumulated in the rumen [18]. Additionally, a linear increase in NH_3_N concentrations as the degree of saturation increased (tallow, partially hydrogenated tallow, hydrogenated tallow, blend of hydrogenated tallow, hydrogenated fatty acids, and hydrogenated fatty acids) has been reported [21]. The study suggested that the dietary triglycerides became more unsaturated, and ruminal protein digestion was inhibited. These results could be related to better synchrony between N and energy availability for microorganism activity. These results did not agree with previous studies where the ruminal NH_3_N concentrations were not affected by supplemental fat or fat source [26,72,73,74].

The cultural pH was lower for the CON-fed fermenter compared to the fat-fed fermenters. A drop in the pH with high-starch diets is common in the literature [75]. The inclusion of fat in the diets increased the cultural pH, and the highest pH values were observed for the CO-fed fermenter, followed by the PF-fed fermenter and SO-fed fermenter compared to the CON-fed fermenter. This agrees with a study conducted by [21], where they reported an increase in the ruminal pH as different saturated fats were fed, and they attributed that to the lower fermentable carbohydrate content in these diets. A study by [76,77] reported a similar rumen pH between treatments as DDGS increased in the diets. In contrast, ref. [70] observed a linear decrease in the rumen pH as DDGS increased in the diets, and they attributed that to the F:C ratio. The E*h* was the lowest for CO-fed fermenter than the other two fat treatments and CON-fed fermenter and the opposite for the rH. The relative H score ranges from 0 to 42, and 28 is the mid-point because lower than 28 is reducing and higher than 28 is oxidizing. A study by [78] stated that there is a relationship between pH and E*h*, and it seemed that the ruminal E*h* moved toward higher E*h* when the pH dropped, which is exactly the case in the present study. We observed that the lowest reducing E*h* (−360.64) was observed when the pH was the highest (6.13) with a CO-fed fermenter. These findings could be related to the rapidly fermentable carbohydrates for the CON-fed fermenter [79].

The total protozoa count was decreased with fat inclusion in the diets, and it was the lowest with the SO-fed fermenter, followed by the PF-fed fermenter and CO-fed fermenter, and then the highest was the CON-fed fermenter. A decrease occurred in total rumen protozoa when fat was supplemented (tallow, partially hydrogenated tallow, and animal-vegetable fat) and as the supplemental fat source became more unsaturated, as in the case of the SO-fed fermenter in the current study [26]. However, ref. [80] observed that the total protozoa count was higher with high-fat diets (5% animal–vegetable fat). They attributed that the direct incorporation of preformed FAs might have spared more energy for cell growth, or the BH would have decreased FA’s toxic concentrations below the threshold. Also, ref. [81] reported an increase in total protozoa counts in diets containing 4% fat from DDGS and monensin. A study by [30] reported that the polyunsaturated fatty acids did not affect total protozoa counts; however, they observed an increase in *Epidinium* genus spp. with high polyunsaturated fatty acid treatment, and they stated that the reason behind that is unclear. A decrease in protozoa when Holstein dairy cows were fed 4% supplemental soybean as a source of linoleic acid and linseed oil as a source of linolenic acid in diets has been reported [82]. Also, they observed a lower number of cellulolytic bacteria and a higher proteolytic bacteria number. Polyunsaturated fatty acids showed a more negative effect than the saturated fatty acids on the metabolism of cellulolytic bacteria and a direct effect on ruminal protozoa [26,83]. Several in vitro and in vivo studies showed a toxic effect of linoleic acid on ruminal protozoa, with a consistent decrease in protozoa counts [84,85,86,87]. In addition, ref. [88] observed that linolenic acid was more toxic to ruminal bacteria than linoleic acid. The effects of fatty acid on bacteria can directly disrupt the microbial cell membrane and lipid coating of bacteria and feed particles and create antimicrobial effects on the bacterial population [11].

**Table 4:** Volatile fatty acids, NH_3_N, pH, E*h*, and protozoa population of continuous culture fermenters fed high-concentrate diets (HC 65%) with high-fat inclusion and different lipid sources (CON 3%, PF 9%, SO 9%, and CO 9% DM).

	Fat Type, * % in the Diet					
Culture Fermentation	CON 3%	PF 9%	SO 9%	CO 9%	SE	*p*-Value
Total VFA, m*M*	111.9 ^a^	83.4 ^b^	88.0 ^b^	66.3 ^c^	4.39	<0.01
Individual VFA, mol/100 mol						
Acetate	49.9 ^a^	47.4 ^ab^	45.2 ^bc^	44.3 ^c^	1.26	<0.01
Propionate	31.4 ^a^	30.2 ^ab^	31.5 ^a^	27.1 ^b^	1.19	0.02
Butyrate	11.8 ^b^	15.7 ^ab^	14.3 ^b^	18.9 ^a^	1.24	<0.01
Valerate	6.05 ^bc^	5.65 ^c^	8.22 ^ab^	8.64 ^a^	0.81	<0.01
Isobutyrate	0.68 ^b^	0.80 ^ab^	0.71 ^ab^	0.98 ^a^	0.12	<0.01
Acetate/propionate	1.62	1.58	1.46	1.66	0.08	0.22
NH_3_N, mg/dL	4.84 ^d^	5.64 ^b^	5.09 ^c^	5.91 ^a^	0.02	<0.01
pH	5.78 ^d^	6.05 ^b^	5.94 ^c^	6.13 ^a^	0.01	<0.01
E*h*, ^1^ mV	−296 ^a^	−265 ^a^	−279 ^a^	−360 ^b^	17.9	<0.01
rH ^2^	8.35 ^a^	9.92 ^a^	9.22 ^a^	6.90 ^b^	0.60	0.01
Protozoa, 10^3^/mL	26.0 ^a^	19.4 ^c^	16.9 ^d^	22.1 ^b^	0.55	<0.01

^1^ E*h* = redox potential; ^2^ rH; Clark’s exponent = ((E*h* + 200)/30) + (2 × pH). * Means in the same row, followed by different superscripts (^a^, ^b^, ^c^, and ^d^), are significantly different (*p* < 0.05).

## 4. Conclusions

Simulating precision feeding with high-concentrate and high-fat inclusion diets with different unsaturated fat sources in the continuous culture fermenter had some effects on ruminal fermentation. The total VFA concentration and protozoa population were decreased while maintaining a higher pH and ammonia concentration in more saturated sources than unsaturated fat sources and control treatments. This study demonstrates that dietary poultry fat inclusion and coconut oil inclusion improved apparent digestibility significantly compared to soybean oil and the control diet. Therefore, we can conclude that saturated fatty acids, as in the by-products of dietary poultry fat, or saturated medium-chain fatty acids, as in coconut oil, can be successfully included in rations for precision-fed dairy heifers up to 6% and reduce the DMI further while improving approximately 15% of all nutrient digestibility.

## Figures and Tables

**Table 1:** Ingredient, chemical composition, and fatty acid profiles of high-concentrate diets (HC 65%) with high-fat inclusion and different lipid sources (CON 3%, PF 9%, SO 9%, and CO 9% DM) fed to continuous culture fermenters.

	Fat Type, % in the Diet			
Ingredient, ^1^ %	CON 3%	PF 9%	SO 9%	CO 9%
Coastal bermudagrass hay	5.00	5.00	5.00	5.00
Whole corn silage	30.0	30.0	30.0	30.0
Ground corn	51.8	40.8	40.8	40.8
Soybean meal (SBM)	11.2	16.4	16.4	16.4
Mineral mix	2.00	2.00	2.00	2.00
Fat inclusion	0.00	5.79	5.79	5.79
**Chemical composition**				
DM %	90.5	90.6	90.7	90.0
OM, %	95.6	95.2	94.8	95.5
CP, %	12.8	14.0	14.2	14.2
Soluble CP, % CP	23.4	24.3	24.3	23.7
NDF, %	20.8	19.8	20.2	20.4
ADF, %	9.84	9.20	9.58	9.71
Sugar, %	3.60	4.10	4.00	3.80
Starch, %	45.2	37.9	38.7	38.4
Ether extract, %	3.52	8.56	8.69	8.31
NSC, %	48.8	42.0	42.7	42.2
NFC, ^2^ %	58.5	52.8	51.7	52.6
TDN	78.9	86.0	85.4	82.4
ME, ^3^ Mcal/Kg	2.88	3.14	3.11	3.01
Ash, %	4.41	4.83	5.18	4.55
**Fatty acid, %**				
C8:0	0.05	0.07	0.03	0.06
C10:0	0.01	0.02	0.01	0.04
C12:0	0.05	0.06	0.02	34.5
C14:0	0.11	0.44	0.07	28.3
C14:1T	0.01	0.01	0.00	0.12
C14:1	0.08	0.08	0.04	0.01
C16:0	14.7	23.9	11.7	6.00
C18:0	0.04	4.59	0.03	0.99
C18:1	25.7	32.2	17.3	7.59
C18:1–11C	1.68	2.65	19.9	0.90
C18:2	51.9	31.3	44.9	18.9
C18:3	4.40	2.26	4.17	1.80
C22:0	0.24	0.30	0.19	0.07
C24:0	0.49	1.25	0.96	0.37
C22:2	0.01	0.34	0.41	0.26
C22:6	0.51	0.51	0.26	0.06
Total, mg/g	28.9	80.2	82.3	78.2

^1^ All diets were ground to 2 mm. ^2^ NFC: non-fiber carbohydrates = 100 − (CP + ether extract + NDF + Ash). ^3^ ME calculated according to [18] using TDN values as reported by Cumberland Valley Analytical Services, Inc., Waynesboro, PA., USA ME = (TDN × 4.409 × 1.01 − 0.45) × 0.82. To represent the increase in energy as fat increased in the diets, ME = (TDN × 4.409 × 1.01 − 0.45) + (0.0046 × (EE − 3) × 0.82 (modified from [18]).

**Table 2:** Nutrient apparent digestibility of continuous culture fermenters fed high-concentrate diets (HC 65%) with high-fat inclusion and different lipid sources (CON 3%, PF 9%, SO 9%, and CO 9% DM).

	Fat Type, * % in the Diet					
Digestibility, %	CON 3%	PF 9%	SO 9%	CO 9%	SE	*p*-Value
DM	69.0 ^c^	80.1 ^a^	76.3 ^b^	80.9 ^a^	0.35	<0.01
OM	74.5 ^c^	84.6 ^a^	81.4 ^b^	85.4 ^a^	0.28	<0.01
NDF	41.8 ^c^	60.0 ^a^	54.0 ^b^	60.7 ^a^	0.65	<0.01
ADF	33.9 ^c^	50.6 ^a^	46.6 ^b^	51.1 ^a^	0.81	<0.01
FA	68.6 ^b^	84.3 ^a^	82.8 ^a^	82.6 ^a^	1.78	0.02
Starch	99.7 ^c^	99.9 ^ab^	99.9 ^b^	99.9 ^a^	0.01	<0.01

* Means in the same row, followed by different superscripts (^a^, ^b^, and ^c^), are significantly different (*p* < 0.05).

**Table 3:** Daily fatty acids flow and biohydrogenation of continuous culture fermenters fed high-concentrate diets (HC 65%) with high-fat inclusion and different lipid sources (CON 3%, PF 9%, SO 9%, and CO 9% DM).

	Fat Type, * % in the Diet					
FA Outflow, mg/d	CON 3%	PF 9%	SO 9%	CO 9%	SE	*p*-Value
**Saturated**						
C8:0	16.5 ^a^	12.8 ^a^	10.4 ^ab^	2.15 ^b^	2.90	0.01
C10:0	3.52 ^a^	1.17 ^b^	1.96 ^ab^	2.32 ^ab^	0.64	0.13
C12:0	1.78 ^b^	2.09 ^b^	1.99 ^b^	641 ^a^	18.9	<0.01
C14:0	2.41 ^b^	8.70 ^b^	3.00 ^b^	476 ^a^	25.8	<0.01
C16:0	145 ^c^	419 ^a^	248 ^b^	72.9 ^c^	27.0	<0.01
C18:0	29.3 ^c^	501 ^a^	233 ^b^	22.3 ^c^	37.9	0.03
C22:0	2.20 ^c^	6.66 ^a^	4.04 ^b^	1.62 ^c^	0.54	0.02
C24:0	2.70 ^b^	4.91 ^ab^	6.18 ^a^	2.36 ^b^	0.97	0.05
**Unsaturated**						
C18:1	237 ^ab^	230 ^b^	316 ^a^	117 ^c^	29.1	0.04
C18:2	434 ^b^	227 ^c^	587 ^a^	241 ^c^	44.9	0.04
C18:3	24.5 ^b^	13.5 ^b^	43.8 ^a^	16.1 ^b^	3.81	0.05
Total	1106 ^b^	2628 ^a^	2609 ^a^	2422 ^a^	102	0.01
**Biohydrogenation, ^1^ %**						
C18:2	42.1 ^c^	78.9 ^a^	63.3 ^b^	62.1 ^b^	3.39	<0.01
C18:3	60.3 ^b^	82.8 ^a^	70.6 ^b^	69.4 ^b^	3.63	0.01

^1^ Expressed as milligrams of input−milligrams of outflow/milligrams of input for 18:2 and 18:3. * Means in the same row, followed by different superscripts (^a^, ^b^, and ^c^), are significantly different (*p* < 0.05).

## Data Availability

Dataset available on request from the authors.

## Appendix A

The nutrient inputs of high-concentrate diets (HC 65%) with high-fat inclusion and different lipid sources (CON 3%, PF 9%, SO 9%, and CO 9% DM) fed to continuous culture fermenters are listed below.

	**Fat Type, % in the Diet**			
**Nutrient Input, g/d**	**CON 3%**	**PF 9%**	**SO 9%**	**CO 9%**
As fed	53.4	47.7	47.7	47.7
DM	48.3	43.2	43.2	42.9
OM	46.1	41.1	41.0	41.0
N	0.99	0.97	0.98	0.98
EE	1.70	3.70	3.76	3.57
NDF	10.0	8.57	8.74	8.74
ADF	4.76	3.98	4.15	4.15
Starch	18.9	13.8	13.7	13.6
NFC	28.2	22.8	22.3	22.5
Ash	2.13	2.09	2.24	1.96
ME, ^1^ Mcal/d	0.12	0.12	0.12	0.12
**FA input, mg/d**				
Total	1401	3465	3563	3360
C8:0	0.72	2.45	0.95	1.95
C10:0	0.14	0.86	0.13	1.45
C12:0	0.72	2.18	0.71	1157
C14:0	1.57	15.3	2.53	951
C16:0	196	811	408	191
C18:0	55	158	96	33.2
C22:0	3.30	10.5	6.84	2.24
C24:0	6.92	43.2	34.3	12.3
C18:1	359	1046	617	255
C18:2	728	1077	1600	637
C18:3	61.6	78.3	148	52.6
**Fractional turnover rate**				
Liquid fraction, %/h	8.60	7.76	7.76	7.76
Solid fraction, %/h	3.84	3.22	3.22	3.22
^1^ ME (Mcal/d) calculated as ME = (digested OM × 4.409 × 1.01 − 0.45) × 0.82. To represent the increase in energy as fat increased in the diets, ME = (digested OM × 4.409 × 1.01 − 0.45) + (0.0046 × (EE − 3) × 0.82 (modified from [18]).				
